# The CpG island methylator phenotype may confer a survival benefit in patients with stage II or III colorectal carcinomas receiving fluoropyrimidine-based adjuvant chemotherapy

**DOI:** 10.1186/1471-2407-11-344

**Published:** 2011-08-10

**Authors:** Byung-Hoon Min, Jeong Mo Bae, Eui Jin Lee, Hong Suk Yu, Young-Ho Kim, Dong Kyung Chang, Hee Cheol Kim, Cheol Keun Park, Suk-Hee Lee, Kyoung-Mee Kim, Gyeong Hoon Kang

**Affiliations:** 1 Departments of Medicine, Samsung Medical Center, Sungkyunkwan University School of Medicine, Seoul, Korea; 2 Department of Pathology, Seoul National University College of Medicine, Seoul, Korea; 3 Department of Pathology, Samsung Medical Center, Sungkyunkwan University School of Medicine, Seoul, Korea; 4 Department of Surgery, Samsung Medical Center, Sungkyunkwan University School of Medicine, Seoul, Korea; 5 Department of Pathology, Daehang Hospital, Seoul, Korea

## Abstract

**Background:**

Colorectal carcinoma (CRC) with CpG island methylator phenotype (CIMP) is recognized as a distinct subgroup of CRC, and CIMP status affects prognosis and response to chemotherapy. Identification of CIMP status in CRC is important for proper patient management. In Eastern countries, however, the clinicopathologic and molecular characteristics and prognosis of CRCs with CIMP are still unclear.

**Methods:**

A total of 245 patients who underwent their first surgical resection for sporadic CRC were enrolled and CIMP status of the CRCs was determined using the quantitative MethyLight assay. The clinicopathologic and molecular characteristics were reviewed and compared according to CIMP status. In addition, the three-year recurrence-free survival (RFS) of 124 patients with stage II or stage III CRC was analyzed in order to assess the effectiveness of fluoropyrimidine-based adjuvant chemotherapy with respect to CIMP status.

**Results:**

CIMP-high CRCs were identified in 34 cases (13.9%), and were significantly associated with proximal tumor location, poorly differentiated carcinoma, mucinous histology, and high frequencies of BRAF mutation, *MGMT *methylation, and MSI-high compared to CIMP-low/negative carcinomas. For patients with stage II or III CIMP-low/negative CRCs, no significant difference was found in RFS between those undergoing surgery alone and those receiving surgery with fluoropyrimidine-based adjuvant chemotherapy. However, for patients with CIMP-high CRCs, patients undergoing surgery with fluoropyrimidine-based adjuvant chemotherapy (n = 17; three-year RFS: 100%) showed significantly better RFS than patients treated with surgery alone (n = 7; three-year RFS: 71.4%) (*P *= 0.022).

**Conclusions:**

Our results suggest that selected patients with CIMP-high CRC may benefit from fluoropyrimidine-based adjuvant chemotherapy with longer RFS. Further large scale-studies are required to confirm our results.

## Background

The epidemiology and clinicopathologic characteristics of colorectal carcinoma (CRC) in Eastern countries differ from those of Western countries in many aspects [1-3]. The distinct lifestyles and diet may underlie these differences [4,5]. Furthermore, recent studies have demonstrated that the molecular pathogenesis of CRC in Eastern countries may be different from that of Western countries [6-8]. The prognosis and response to chemotherapy of patients with CRC may be affected by molecular characteristics [9-11], and it has been suggested that CRC treatment plans should be developed based on individual molecular characteristics. As such, identification of molecular characteristics associated with CRC has important clinical implications.

In addition to chromosomal instability and microsatellite instability (MSI), CpG island methylator phenotype (CIMP), which is characterized by the simultaneous methylation of multiple CpG islands, is currently recognized as one of the major mechanisms underlying colorectal carcinogenesis [12-14]. In this phenotype, widespread methylation of CpG islands results in the epigenetic inactivation of tumor suppressor genes by promoter methylation. CIMP-positive CRCs have been reported to have distinct clinicopathologic profiles compared to their CIMP-low/negative counterparts; older age, female sex, proximal tumor location, poorly differentiated or mucinous histology, and high rates of MSI and *BRAF *mutation [12,13,15-20]. In addition, although some controversies still exist, several studies reported that CIMP status influences prognosis and response to chemotherapy in patients with CRC [21-24]. A previous study reported that CIMP positivity appears to result in improved survival among patients receiving 5-fluorouracil-based adjuvant chemotherapy for stage III CRC [22]. However, a recent large-scale study failed to demonstrate CIMP positivity as a significant prognostic factor in stage II and III CRCs treated with adjuvant chemotherapy [25]. In Eastern countries, few studies of the clinicopathologic features in CIMP-positive CRCs have been conducted [26,27], and patient outcomes with respect to CIMP status have never been explored by quantitative methylation analyses.

In order to determine the clinicopathologic and molecular characteristics and recurrence-free survival (RFS) of CRC according to CIMP status, we selected Korean patients with MSI-high and MSI-low CRCs and analyzed their CIMP status by quantitative methylation analyses. The results of this study will contribute to the determination of clinicopathologic and molecular characteristics and prognoses of CRCs stratified by CIMP status in Korean patients.

## Methods

### Patients and tumor specimens

In order to compare methylation profiles according to MSI status, 49 MSI-high carcinomas and 196 microsatellite stable (MSS) or MSI-low carcinomas were selected from archived samples. All 245 selected patients had undergone their first surgical resection for CRC at Samsung Medical Center (n = 132; random selection after acquisition of 40 MSI-high carcinomas) or Seoul National University Hospital (n = 113; consecutively selected) between July 2003 and October 2006.

Patients with inflammatory bowel disease, a family history of Lynch syndrome or familial adenomatous polyposis, or synchronous multiple colorectal cancers were excluded. Surgical resection techniques were standardized in the respective hospitals during the study period. Tumor stage and depth of invasion were defined according to the tumor, node, metastasis (TNM) classification developed by the American Joint Committee on Cancer [28]. Stage I was assigned in 27, II in 91, III in 82 and IV in 45 cases. None of the patients had received chemotherapy or radiotherapy prior to surgery. During study period, adjuvant chemotherapy regimen for stage II and III CRCs was mainly based on fluoropyrimidines (5-FU or capecitabine). Mayo Clinic regimen was used for administration of 5-FU: six cycles of rapid intravenous infusion of 20 mg/m^2 ^leucovorin, followed immediately by an intravenous bolus of 425 mg/m^2 ^fluorouracil, on days 1 to 5 every four weeks. The palliative chemotherapy regimen for stage IV CRCs was based on fluoropyrimidines, oxaliplatin, or irinotecan.

Formalin-fixed, paraffin-embedded tissues were used for DNA extraction. DNA was extracted from 10 μm sections stained with 0.1% methylene blue by manual microdissection under microscopy using 20-gauge needles and proteinase K solution as described previously [29]. Clinicopathologic data were collected by review of medical records and pathology slides. In the present study, tumors located from the cecum to the transverse colon were classified as proximal and those from the splenic flexure to the rectum were classified as distal.

All patients gave informed consent prior to specimen collection according to our institutional guidelines. This study was carried out in accordance with the Declaration of Helsinki. The institutional review boards at Samsung Medical Center and Seoul National University Hospital approved the study protocol.

### Survival analyses

RFS data for patients with stage I, II or III CRCs was obtained by review of medical records using the intranet resources at both hospitals. RFS was measured from the date of resection to the date of the first recurrence documented by imaging or pathologic confirmation or until the censoring date of May 31, 2010.

We performed a subgroup analysis of RFS to determine the impact of fluoropyrimidine-based adjuvant chemotherapy on clinical outcome in patients with stage II and stage III CRCs with respect to CIMP status. Among the 173 patients with stage II or stage III CRCs, we excluded from analysis 20 patients who received oxaliplatin-based adjuvant chemotherapy, 15 patients with rectal cancers who received concurrent chemo-radiation therapy and 14 patients who did not undergo any follow-up examinations. A total of 124 patients with stage II or stage III CRCs were ultimately analyzed. Among them, 46 cases had undergone surgery without subsequent adjuvant chemotherapy and 78 received fluoropyrimidine-based adjuvant chemotherapy after surgery. The median follow-up time for these patients was 44.5 months (range, 6-75 months).

### Molecular analyses for CIMP

Five widely used methylation markers that allow for excellent discrimination of CIMP status were employed in this study. This panel of markers consists of the *CACNA1G, IGF2, NEUROG1, RUNX3*, and *SOCS1 *genes [20]. The MethyLight assay (Applied Biosystems) was applied to quantitatively determine methylation status as previously described [20]. Briefly, two sets of primers and probes designed specifically for bisulfite-converted DNA were used. Reaction specificity for methylated DNA was confirmed separately using human DNA treated with CpG methyltransferase SssI (New England Biolabs). In addition to CIMP markers, methylation of *O^6^-methylguanine-DNA methyltransferase *(*MGMT*) gene was also analyzed by methylation-specific polymerase chain reaction due to its importance in KRAS mutations [30-32] and clinical significance in survival [33]. CIMP classification was based on the numbers of methylated genes in each panel. Tumors were classified as CIMP-high if three or more markers were methylated, CIMP-low if one or two markers were methylated, and CIMP-negative if a methylated marker was not observed [14,20].

### Molecular analyses of MSI status and the presence of BRAF and KRAS mutations

We used five microsatellite markers recommended by the National Cancer Institute Workshop on MSI to determine MSI status [34]. PCR analyses were performed using a DNA autosequencer (Applied Biosystems 373A sequencer; Applied Biosystems, CA, USA). The mobility shift of PCR products from the tumor DNA was compared to that from corresponding normal colonic mucosa. Tumors were classified as MSI-high if band shifts were observed in two or more markers compared to the control, MSI-low if shifts were observed in one marker, and MSS if no shift was observed [34]. *BRAF *mutations in exon 11 and exon 15 and *KRAS *mutations in codons 12 and 13 in exon 2 were analyzed by PCR and automated sequencing as previously described [29].

### Statistical analysis

Patient clinicopathologic features were compared using the *x*^2 ^test and Student's *t*-test, with *p *< 0.05 considered significant. Survival curves were analyzed by the Kaplan-Meier method, and differences between individual curves were evaluated by the log-rank test. Multivariate analysis using the Cox proportional hazards model was performed to explore the potential association between clinicopathologic and molecular parameters and RFS.

## Results

### Clinicopathologic and molecular characteristics according to the CIMP status

The characteristics of CIMP-high and CIMP-low/negative CRCs are summarized in Table 1. CIMP-high carcinomas were found in 34 cases (13.9%) and were more frequently associated with proximal tumor locations, poorly differentiated histology, mucinous histology, *BRAF *mutation, *MGMT *methylation, and MSI-high compared to CIMP-low/negative carcinomas (*P *< 0.05).

**Table 1:** Clinicopathologic and molecular characteristics of colorectal cancers according to the CIMP status

	CIMP-low/negative (n = 211)	CIMP-high (n = 34)	*P *value
Age (yrs)			0.388
Mean ± SD	59.7 ± 11.9	61.6 ± 11.9	
Median (range)	62.0 (29 - 83)	63.5 (33 - 82)	
Gender (%)			0.752
Male	118 (55.9)	20 (58.8)	
Female	93 (44.1)	14 (41.2)	
Tumor site (%)			< 0.001
Proximal	64 (30.3)	**23 (67.6)**	
Distal	147 (69.7)	11 (32.4)	
Lymph node metastasis (%)			0.770
Absent	106 (50.2)	18 (52.9)	
Present	105 (49.8)	16 (47.1)	
AJCC stage (%)			0.548
I/II	100 (47.4)	18 (52.9)	
III/IV	111 (52.6)	16 (47.1)	
Differentiation (%)			< 0.001
Well/Moderate	191 (90.5)	21 (61.8)	
Poor/Mucinous	20 (9.5)	**13 (38.2)**	
Mucinous histology (%)			0.045...
Absent	202 (95.7)	28 (82.4)	
Present	9 (4.3)	**6 (17.6)**	
BRAF (%)			< 0.001
Wild type	209 (99.1)	25 (73.5)	
Mutation	2 (0.9)	**9 (26.5)**	
KRAS (%)			0.894
Wild type	139 (65.9)	22 (64.7)	
Mutation	72 (34.1)	12 (35.3)	
MGMT methylation (%)			< 0.001
Absent	153 (72.5)	12 (35.3)	
Present	58 (27.5)	**22 (64.7)**	
MSI status (%)			< 0.001
MSS/MSI-low	183 (86.7)	13 (38.2)	
MSI-high	28 (13.3)	**21 (61.8)**	

*CIMP *CpG island methylator phenotype, *AJCC *American Joint Committee on Cancer, *MSI *microsatellite instablity, *MSS *microsatellite stable.

Bold numbers indicate features associated with CIMP-high colorectal cancers *p *values < 0.05 were adjusted with Bonferroni correction to correct for potential false positive results

All *BRAF *mutations were missense mutations in codon 600 of exon 15 (V600E) and *BRAF *and *KRAS *mutations were mutually exclusive. *BRAF *mutations were found more frequently in CIMP-high (26.5%) than in CIMP-low/negative carcinomas (0.9%). When stratified by MSI status, *BRAF *mutation rates were 12.2% for MSI-high CRCs and 2.6% for MSI-low/MSS CRCs (*P *= 0.010). The rates of *KRAS *mutations were comparable in CIMP-high (35.3%) and CIMP-low/negative carcinomas (34.1%). When stratified by MSI status, *KRAS *mutation rates were 28.6% for MSI-high CRCs and 35.7% for MSI-low/MSS CRCs (*P *= 0.346). *KRAS *mutation was significantly more prevalent in carcinomas with *MGMT *methylation (46.3%) than in cases without *MGMT *methylation (46.3% vs. 28.5%; *P *= 0.006).

### Survival analyses in patients with stage I, II, and III CRCs

There were a total of 200 patients with stage I, II, and III CRCs, and the three-year RFS survival for each stage was 91.6%, 88.0%, and 71.6%, respectively. In these patients, RFS did not differ significantly between CIMP-high and CIMP-low/negative groups (three-year RFS: 89.0% vs. 80.6%; *P *= 0.274). Regarding MSI, patients with MSI-high CRCs showed longer RFS than those with MSI-low or MSS CRCs, although this difference did not reach statistical significance (three-year RFS: 90.6% vs. 79.2%; *P *= 0.069).

On multivariate analysis of RFS of patients with stage I, II or stage III CRCs, TNM stage was an independent predictor of recurrence (Table 2). We performed an additional multivariate analysis of RFS for the 124 patients with stage II or stage III CRCs who had undergone surgery only or received fluoropyrimidine-based adjuvant chemotherapy after surgery. The following variables were included in the multivariate model: age, gender, TNM stage, histological differentiation, CIMP status, MSI status, treatment modality (surgery alone versus surgery with chemotherapy), *BRAF *mutation, *KRAS *mutation and *MGMT *methylation. Only TNM stage was identified as an independent predictor of RFS in these patients (hazard ratio: 5.963, 95% CI: 1.351-26.314). As no recurrence occurred in patients with CIMP-high CRCs treated with fluoropyrimidine-based adjuvant chemotherapy, the effect of an interaction between CIMP status and treatment modality in these patients could not be analyzed using the Cox proportional hazards model.

**Table 2:** Multivariate analysis of recurrence-free survival in patients with stage I-III colon cancers

		Univariate HR (95% CI)	Multivariate HR (95% CI)
Age		1.015 (0.985-1.047)	1.028 (0.995-1.062)
Sex	Male	1 (referent)	1 (referent)
	Female	1.438 (0.733-2.819)	1.600 (0.799-3.204)
Stage	Stage I/II	1 (referent)	1 (referent)
	Stage III	2.303 (1.163-4.561)	2.587 (1.267-5.281)
Differentiation	Well/Moderate	1 (referent)	1 (referent)
	Poor/Mucinous	1.210 (0.426-3.435)	1.347 (0.415-4.375)
CIMP	CIMP-low/negative	1 (referent)	1 (referent)
	CIMP-high	0.523 (0.160-1.710)	0.806 (0.207-3.136)
MSI	MSI-low/MSS	1 (referent)	1 (referent)
	MSI-high	0.394 (0.139-1.118)	0.485 (0.139-1.701)
BRAF mutation	No	1 (referent)	1 (referent)
	Yes	No recurrence	No recurrence
KRAS mutation	No	1 (referent)	1 (referent)
	Yes	1.096 (0.542-2.214)	1.185 (0.568-2.471)
*MGMT *methylation	No	1 (referent)	1 (referent)
	Yes	0.667 (0.311-1.429)	0.703 (0.304-1.628)

*HR *hazard ratio, *CIMP *CpG island methylator phenotype, *MSI *microsatellite instability, *MSS *microsatellite stable

### Survival analyses according to CIMP status and treatment modality in patients with stage II or III CRCs

To explore the impact of fluoropyrimidine-based adjuvant chemotherapy for stage II and stage III CRCs according to CIMP status, we analyzed the RFS of 124 patients with stage II or stage III CRCs. The RFS was not significantly different between CIMP-high group and CIMP-low/negative group (three-year RFS: 91.7% vs. 84.0%; *P *= 0.31). In addition, RFS was not significantly affected by treatment modality. The three-year RFS for patients who received surgery alone and those who underwent surgery followed by fluoropyrimidine-based adjuvant chemotherapy was 84.1% and 86.5%, respectively (*P *= 0.387). Patients with MSI-high CRCs experienced longer RFS compared to those with MSI-low or MSS CRCs, although this difference did not reach statistical significance (three-year RFS: 94.0% vs. 82.3%; *P *= 0.088).

**Figure 1:**
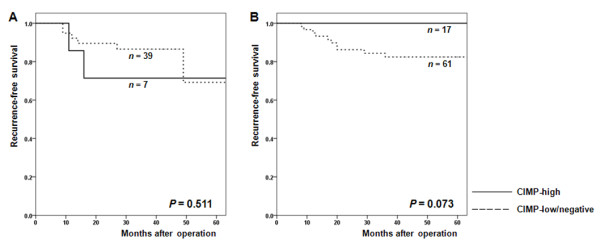
**Recurrence-free survival (RFS) of stage II-III colorectal cancer patients according to the CpG island methylation phenotype (CIMP)**. (A) Kaplan-Meier curves for RFS of patients treated by surgery alone. The three-year RFS for CIMP-high and CIMP-low/negative groups was 71.4% and 86.5%, respectively. (B) Kaplan-Meier curves for the RFS of patients treated with surgery and fluoropyrimidine-based adjuvant chemotherapy. The three-year RFS for CIMP-high and CIMP-low/negative groups was 100% and 82.4%, respectively.

Figure 1 shows RFS according to CIMP status in patients who underwent surgery alone (Figure 1A) and in patients who received fluoropyrimidine-based adjuvant chemotherapy after surgery (Figure 1B) for stage II or III CRCs. In patients treated with surgery alone (Figure 1A), RFS did not differ significantly between CIMP-high and CIMP-low/negative groups. However, in patients treated with surgery and fluoropyrimidine-based adjuvant chemotherapy (Figure 1B), CIMP-high group had a longer RFS than the CIMP-low/negative group although this difference did not reach statistical significance (three-year RFS: 100% vs. 82.4%; *P *= 0.073).

**Figure 2:**
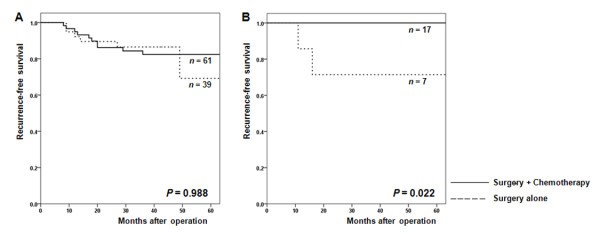
**Recurrence-free survival (RFS) of stage II-III colorectal cancer patients according to treatment**. (A) Kaplan-Meier curves for the RFS of patients with the CpG island methylation phenotype (CIMP)-low/negative CRCs. The three-year RFS for patients treated by surgery with adjuvant chemotherapy and those treated by surgery alone was 82.4% and 86.5%, respectively. (B) Kaplan-Meier curves for the RFS of patients with CIMP-high CRCs. The three-year RFS for the surgery with adjuvant chemotherapy group and the surgery alone group was 100% and 71.4%, respectively.

**Figure 3:**
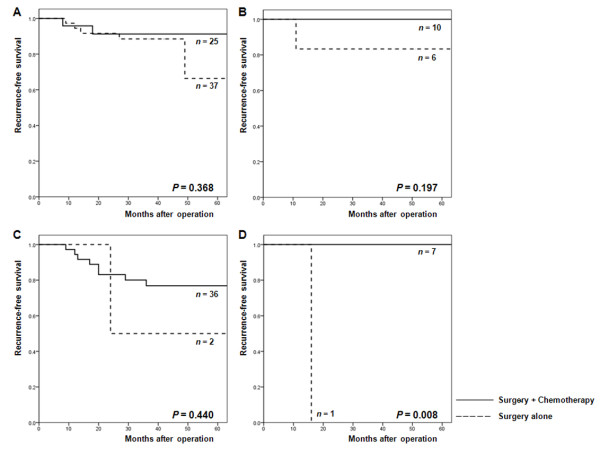
**Recurrence-free survival (RFS) according to treatment in patients with stage II and stage III colorectal cancers**. (A) Kaplan-Meier curves for the RFS of patients with stage II CpG island methylation phenotype (CIMP)-low/negative CRCs. (B) Kaplan-Meier curves for the RFS of patients with stage II CIMP-high CRCs. (C) Kaplan-Meier curves for the RFS of patients with stage III CIMP-low/negative CRCs. (D) Kaplan-Meier curves for the RFS of patients with stage III CIMP-high CRCs.

Since our results suggested an interaction between CIMP-high status and fluoropyrimidine-based chemotherapy in stage II or III CRCs, we performed survival analyses according to treatment modality in patients with CIMP-low/negative (Figure 2A) and CIMP-high CRCs (Figure 2B). The characteristics of these patients according to CIMP status and treatment modality are summarized in Table 3. In both patients with CIMP-low/negative and patients with CIMP-high CRCs, those undergoing surgery followed by chemotherapy showed a higher LN metastasis rate and consequently a more advanced TNM stage than those undergoing surgery alone (*P *< 0.001 for CIMP-low/negative CRCs; *P *= 0.352 for CIMP-high CRCs). In patients with stage II or III CIMP-low/negative CRCs (Figure 2A), RFS was not significantly different between those undergoing surgery alone and those receiving surgery with adjuvant chemotherapy. When stratified according to TNM stage, a similar trend was observed for both stage II and stage III CRCs (Figure 3A and 3C). However, in patients with stage II or III CIMP-high CRCs (Figure 2B), the RFS of those treated with surgery and fluoropyrimidine-based adjuvant chemotherapy was significantly higher than that of patients treated with surgery alone (three-year RFS: 100% vs. 71.4%; *P *= 0.022). Figures 3B and 3D show the RFS for patients with stage II and stage III CIMP-high CRCs, respectively. In both stages, patients receiving adjuvant chemotherapy demonstrated longer RFS than those receiving surgery alone.

**Table 3:** Clinicopathologic and molecular characteristics of colorectal cancers in patients with stage II or stage III colorectal cancers who underwent surgery alone or received fluoropyrimidine-based adjuvant chemotherapy

	CIMP-low/negative Surgery alone (n = 39)	CIMP-low/negative Surgery + CRx (n = 61)	CIMP-high Surgery alone (n = 7)	CIMP-high Surgery + CRx (n = 17)
Age (yrs)				
Mean ± SD	63.4 ± 11.0	58.7 ± 10.8	72.9 ± 7.1	60.1 ± 10.7
Median (Range)	66.0 (42 - 83)	64.0 (36 - 75)	75.0 (63 - 82)	63.0 (33 - 72)
Gender (%)				
Male	23 (59.0)	34 (55.7)	3 (42.9)	11 (64.7)
Female	16 (41.0)	27 (44.3)	4 (57.1)	6 (35.3)
Tumor site (%)				
Proximal	14 (35.9)	19 (31.1)	6 (85.7)	12 (70.6)
Distal	25 (64.1)	42 (68.9)	1 (14.3)	5 (29.4)
Lymph node metastasis (%)				
Absent	37 (94.9)	25 (41.0)	6 (85.7)	10 (58.8)
Present	2 (5.1)	36 (59.0)	1 (14.3)	7 (41.2)
AJCC stage (%)				
II	37 (94.9)	25 (41.0)	6 (85.7)	10 (58.8)
III	2 (5.1)	36 (59.0)	1 (14.3)	7 (41.2)
Differentiation (%)				
Well/Moderate	34 (87.2)	56 (91.8)	6 (85.7)	9 (52.9)
Poor/Mucinous	5 (12.8)	5 (8.2)	1 (14.3)	8 (47.1)
Mucinous histology (%)				
Absent	36 (92.3)	59 (96.7)	7 (100.0)	12 (70.6)
Present	3 (7.7)	2 (3.3)	0 (0.0)	5 (29.4)
BRAF (%)				
Wild type	39 (100.0)	61 (100.0)	4 (57.1)	14 (82.4)
Mutation	0 (0.0)	0 (0.0)	3 (42.9)	3 (17.6)
KRAS (%)				
Wild type	22 (56.4)	45 (73.8)	3 (42.9)	11 (64.7)
Mutation	17 (43.6)	16 (26.2)	4 (57.1)	6 (35.3)
MGMT methylation (%)				
Absent	26 (66.7)	47 (77.0)	3 (42.9)	5 (29.4)
Present	13 (33.3)	14 (23.0)	4 (57.1)	12 (70.6)
MSI status (%)				
MSS/MSI-low	33 (84.6)	50 (82.0)	4 (57.1)	3 (17.6)
MSI-high	6 (15.4)	11 (18.0)	3 (42.9)	14 (82.4)

*CIMP *CpG island methylator phenotype, *CRx *Fluoropyrimidine-based adjuvant chemotherapy

*MSI *microsatellite instablity, *MSS *microsatellite stable

## Discussion

CIMP is recognized as one of the major pathways in colorectal carcinogenesis [12-14]. Recent studies have demonstrated that the molecular pathogenesis of CRCs may differ according to ethnic background [6-8]. Studies of CIMP CRCs in Eastern countries are rare and the relationship between CIMP and survival or treatment modality has not been described [26,27]. CIMP-high CRCs were found to be associated with older age, female sex, proximal tumor location, poorly differentiated or mucinous histology, and higher frequencies of MSI-high and *BRAF *mutation [12,13,15-20], and nearly all of these characteristics are similar to those of MSI-high carcinomas [34,35]. In the present study, we also found that CIMP-high CRCs were more frequently associated with proximal tumor location, poorly differentiated histology, mucinous histology, *BRAF *mutation, and MSI-high status compared to their CIMP-low/negative counterparts.

Although most of the clinicopathologic features associated with CIMP are consistent with previous reports, the frequency of *BRAF *mutations observed in the present study (26.5% of CIMP-high CRCs) was much lower than that reported by studies of patients from Western populations (60-70% of CIMP-high CRCs) (Table 4) [20,36,37]. These same studies found *BRAF *mutation frequencies ranging from 48.6% to 76.1% in MSI-high CRCs (Table 3) [20,36,37]. In the present study, however, BRAF mutation rate was only 12.2% for MSI-high CRCs, which was consistent with a previous report for patients of the same ethnic background (10.5% among MSI-high carcinomas) [27]. Recently, we performed a study directly comparing the molecular features of sessile serrated adenoma, which is a potential precursor of CIMP-high CRCs, from Korean and Western populations. In that study, both *BRAF *mutation (40% versus 80%) and *hMLH1 *methylation (25% versus 45%) found in sessile serrated adenomas were less frequent in Korea than in the USA. Therefore, it is possible that the lower rate of *BRAF *mutation observed in CIMP-high CRCs in Korea is established from the precursor level [38].

**Table 4:** BRAF mutation and KRAS mutation rates according to CIMP and MSI status

	Present study		**Lee et al. **[27]		**Weisenberger et al. **[20]		**Ogino et al. **[37]		**Kambara et al. **[36]	
	
	BRAF (%)	KRAS (%)	BRAF (%)	KRAS (%)	BRAF (%)	KRAS (%)	BRAF (%)	KRAS (%)	BRAF (%)	KRAS (%)
CIMP-low/negative	0.9	34.1	1.1	31.5	1.3	35.4	6.2	41.6	10.3	37.2
CIMP-high	26.5	35.3	11.9	38.1	72.7	9.7	61.2	7.5	76.9	15.4
MSI-low/MSS	2.6	35.7	3.4				8.9	41.3	8.6	44.4
MSI-high	12.2	28.6	10.5				48.6	10.0	76.1	2.2

*CIMP *CpG island methylator phenotype, *MSI *microsatellite instability, *MSS *microsatellite stable

CIMP status reportedly affects the prognosis and response to chemotherapy in patients with CRC [21-24]; however, the effect of CIMP status on prognosis is still controversial. A previous study reported that CIMP-high carcinomas appeared to result in improved survival among patients who received 5-fluorouracil-based adjuvant chemotherapy [22]. In contrast, other studies reported worse survival in CIMP-high tumors compared to CIMP-low/negative carcinomas [23,25]. In this first study with different ethnic background, we found that patients with CIMP-high CRC treated with surgery and fluoropyrimidine-based adjuvant chemotherapy demonstrated significantly longer RFS than did CIMP-high CRC patients treated with surgery alone despite their higher frequency of LN metastasis and more advanced TNM stage. These observations are consistent with the previous study[22]. Because MSI-high patients show better survival compared to MSS/MSI-low patients [34,35], the observed better survival in patients with CRC treated with surgery and adjuvant chemotherapy may have been affected by the higher frequency of MSI-high CRCs in this patient group (82.4% versus 42.9%). So, additional large-scale studies are required to confirm our results.

The relationship between CIMP-high CRCs and responsiveness to 5-FU-based chemotherapy has also been reported [22,39]. Intracellular folate concentrations are known to be critically important in determining response to 5-FU [40,41]. As CIMP-high CRCs show higher levels of 5-10-methylene tetrahydrofolate (CH_2_FH_4) _and FH_4 _compared to CIMP-low/negative CRCs [42], the higher level of folate in CIMP-high CRCs may be a plausible explanation for better response to 5-FU in these patients. Additionally, γ-glutamyl hydrolase, an enzyme that removes glutamates and lowers intracellular folate levels, is expressed in significantly lower levels in CIMP-high CRCs compared to CIMP-low/negative CRCs [43,44], and reduced expression of γ-glutamyl hydrolase is associated with a favorable response to 5-FU-based chemotherapy in patients with metastatic CRCs [45]. Moreover, methylation-induced silencing of dihydropyrimidine dehydrogenase, the rate-limiting enzyme in 5-FU degradation, may also provide a link between CIMP-high CRCs and favorable response to 5-FU [46-48].

The present study had several limitations. The sample size for the RFS analysis was small and there is the potential for selection bias because the cases were neither prospectively recruited nor randomized to receive adjuvant chemotherapy. In addition, the selection of CRCs according to MSI status may represent an additional source of bias in this study. Despite these limitations, we were able to demonstrate that the clinicopathologic and molecular characteristics were distinct and prognoses were different between the methylation subgroups. A large-scale, prospective, randomized study would be ideal to definitively determine the impact of fluoropyrimidine-based adjuvant chemotherapy for stage II and stage III CRCs with respect to CIMP status. However, it is practically impossible to perform this kind of study as adjuvant chemotherapy is now recognized as a standard therapy and patients with stage II and III CRCs not receiving chemotherapy are very rare. In addition, oxaliplatin-based chemotherapy such as FOLFOX has recently replaced fluoropyrimidine-based chemotherapy as the standard adjuvant chemotherapy regimen.

## Conclusions

Our results confirm that CIMP-high CRCs demonstrate distinct clinicopathologic and molecular characteristics even in a different ethnic background from Western countries. In addition, our results indicate that CIMP-high tumors appear to benefit from fluoropyrimidine-based adjuvant chemotherapy. Confirmation of these findings with additional large-scale studies may lead to the improved selection of CRC patients to receive fluoropyrimidine-based adjuvant chemotherapy.

## List of Abbreviations

CIMP: CpG island methylator phenotype; CRC: colorectal carcinoma; CH_2_FH_4_: 5-10-methylene tetrahydrofolate; MSI: microsatellite instability; MSS: microsatellite stable.

## Declaration of Competing interests

The authors declare that they have no competing interests.

## Authors' contributions

BHM designed the study, analyzed the data and drafted the manuscript. JMB and HSY collected and analyzed the data. EJL carried out the molecular genetic studies. YHK, DKC, and HCK provided the samples. CKP and SHL reviewed the pathology specimens. KMK designed the study, reviewed the pathology specimens, and critically revised the manuscript. GHK designed the study, reviewed the pathology specimens and critically revised the manuscript. All authors read and approved the final manuscript.

## Pre-publication history

The pre-publication history for this paper can be accessed here:

http://www.biomedcentral.com/1471-2407/11/344/prepub
